# Supplementary material for: A new method based on physical patterns to impute aerobiological datasets
Source: PLoS One. 2024 Nov 19;19(11):e0314005. doi: 10.1371/journal.pone.0314005 (PMC11575811; doi:10.1371/journal.pone.0314005)
Supplement: Attachment — Submitted filename: Tagliaferro_rev_PONE-D-24-22455_reviewer.pdf [file pone.0314005.s001.pdf]

Tagliaferro, S., Corrochano, A., Marchetti, P., Marcon, A., Le Clainche, S., 2024: A new method based on physical patterns to impute aerobiological datasets. PLOS ONE;

The authors conducted a novel simulation study to evaluate the effectiveness of Gappy Singular Value Decomposition (GSVD), as a data-driven approach, comparing it with the moving mean interpolation, as a statistical approach. However, high variability in pollen concentrations and the increasing number of missing data and the increasing lack of those around the sample mean negatively affected imputation accuracy.

My comments are as follows.

Comments:

- Line 123: You used the 95% method (start: 2.5th percentile; end: 97.5th percentile) when calculating the start and end dates of the pollen season for each pollen type.
  However, if pollen concentrations and within-season distribution vary by year, such an approach is likely to contract or expand season duration independently from climate change. For example, if the API was 2000 for a given year, the start of the pollen season would be when the cumulative concentration reached 50; but if the API for the same location was 5000 for the following year, then the pollen season would not start until the cumulative concentration reached 125. Use of this system to mark the start and end of the pollen season would mask any climate-related or temperature-related changes associated with pollen season duration.
  Therefore, rather than using start and end percentages, to determine the pollen season, it is widely used the first (last) date on which at least 1 pollen grain m^{-3} of air is recorded and at least 5 consecutive (preceding) days also show 1 or more pollen grains m^{-3} (Makra et al., 2012).

  Reference

  Makra, L., Matyasovszky, I., Bálint, B., 2012: Association of allergic asthma emergency room visits with the main biological and chemical air pollutants. Science of the Total Environment, 432, 288-296. doi:10.1016/j.scitotenv.2012.05.088

- Line 136: Graminaceae is the former name of the grass family. I recommend using Poaceae everywhere in the manuscript instead.
- Line 247: correctly: “For both pollen types”, instead of “For both pollens”;
- Line 341: correctly: “with”, instead of “whit”;

Reviewer
