# Supplementary material for: A new method based on physical patterns to impute aerobiological datasets
Source: PLoS One. 2024 Nov 19;19(11):e0314005. doi: 10.1371/journal.pone.0314005 (PMC11575811; doi:10.1371/journal.pone.0314005)
Supplement: Attachment — Submitted filename: Response to Reviewers.docx [file pone.0314005.s002.docx]

Ms. Ref. No.: PONE-D-24-22455

Title: A new method based on physical patterns to impute aerobiological datasets

PLOS ONE

Response to Reviewers

We thank the Editor and the Reviewers for the time and effort dedicated to reviewing. Besides the implementation of the Reviewers’ suggestions, we further improved the reproducibility and dissemination of the new methodology presented in the manuscript by creating a dedicated section on the Modelflows-app website (<https://modelflows.github.io/modelflowsapp/airpollution/>), where we have included a brief overview of the manuscript, along with a video explanation of the methodology. Moreover, upon acceptance for publication, we will upload the original pollen dataset, R code for missing data generation, Python code for imputation, and sample gappy datasets making them available for download. The Editor and Reviewers can find these materials enclosed in the present resubmission as files "for peer review only".

Comments from the Reviewer:

Reviewer: 1

Comments to the Author

This study (A new method based on physical patterns to impute aerobiological datasets) includes an analysis of the effectiveness of Gappy Singular Value Decomposition (GSVD) in aerobiological datasets. It addresses a clear and evident need in the field of aerobiology: the lack of data on pollen concentration on some days. I would like to congratulate the authors of the paper because it is very well written. Thus, I consider that this paper, after minor corrections, is suitable for publication in PLOS ONE. Some minor suggestions are listed below.

**Reply**: we would like to thank the Reviewer for their positive view and suggestions to improve our manuscript. We have revised the manuscript adding some sentences on further studies needed and corrected the points raised by the Reviewer.

We hope that, after the revision, the manuscript will be considered suitable for publication.

1. L177-179: Use roman numerals i and ii without italics.

**Reply**: as suggested by the Reviewer, we have removed the italics format.

2. L322: While reading this paragraph, as a researcher, I would opt for the "simpler" method. Perhaps add that more studies applying GSVD on different pollen types and environments are needed to reach a more generalized conclusion.

**Reply**: we recognize the Reviewer's thoughts regarding the leaning towards a simpler method. As suggested by the Reviewer, this is surely the first application of the GSVD method on aerobiological datasets, and further studies are needed. For this reason, we have added two sentences in the Discussion (Page 14, lines from 322 to 323 of the tracked manuscript) and in the Conclusions concerning the Reviewer's suggestion (Page 16, lines from 372 to 374 of the tracked manuscript):

“However, more applications of the GSVD method on aerobiological data are needed to evaluate its effectiveness across diverse pollen types and environmental conditions.”

“Additional research is required to investigate the application of the GSVD method across diverse pollen types and environmental conditions to draw a definitive conclusion.”

3. L327: Emphasize the importance of linking pollen data with meteorological data, as has been observed, for example, in this study https://doi.org/10.1016/j.scitotenv.2021.145426

**Reply**: we thank the Reviewer for the suggestions. We have added sentences highlighting the importance of meteorological factors in the Discussion (Page 15, lines from 333 to 335 of the tracked manuscript) and at the end of the Conclusions (Page 16, lines from 374 to 375 of the tracked manuscript):

“Meteorological factors, particularly temperature and precipitation, are widely acknowledged to have the greatest influence on pollen variability, affecting both phenological phases and pollen behaviour in the atmosphere [29,30]” (ref. 29: Blanco-Alegre et al., 2021, STOTEN; ref. 30: Schramm et al., 2021, Int J Biometeorol)

“Furthermore, incorporating meteorological data into pollen datasets should be considered to improve imputation accuracy.”

Reviewer: 2

Comments to the Author

The authors conducted a novel simulation study to evaluate the effectiveness of Gappy Singular Value Decomposition (GSVD), as a data-driven approach, comparing it with the moving mean interpolation, as a statistical approach. However, high variability in pollen concentrations and the increasing number of missing data and the increasing lack of those around the sample mean negatively affected imputation accuracy.

**Reply**: we thank the Reviewer for the time and effort dedicated to reviewing and for the suggestions to improve our manuscript. We appreciated their insightful comments and acknowledged the criticisms. We have answered the issue raised by the Reviewer, and we have revised the manuscript according to their suggestions.

We hope that, after the revision, the manuscript will be considered suitable for publication.

1. Line 123: You used the 95% method (start: 2.5th percentile; end: 97.5th percentile) when calculating the start and end dates of the pollen season for each pollen type.
However, if pollen concentrations and within-season distribution vary by year, such an approach is likely to contract or expand season duration independently from climate change. For example, if the API was 2000 for a given year, the start of the pollen season would be when the cumulative concentration reached 50; but if the API for the same location was 5000 for the following year, then the pollen season would not start until the cumulative concentration reached 125. Use of this system to mark the start and end of the pollen season would mask any climate-related or temperature-related changes associated with pollen season duration.
Therefore, rather than using start and end percentages, to determine the pollen season, it is widely used the first (last) date on which at least 1 pollen grain m−3 of air is recorded and at least 5 consecutive (preceding) days also show 1 or more pollen grains m−3 (Makra et al., 2012).

Reference
Makra, L., Matyasovszky, I., Bálint, B., 2012: Association of allergic asthma emergency room visits with the main biological and chemical air pollutants. Science of the Total Environment, 432, 288-296. doi:10.1016/j.scitotenv.2012.05.088

**Reply**: we thank the Reviewer for raising this issue. While various methodologies have been identified in the literature for calculating the pollen season, no "gold standard" method has been established (Steckling-Muschack et al., 2021, Aerobiologia; Tasioulis et al., 2022, Aerobiologia). We agree with the Reviewer that, in analyses assessing temporal trends such as those on the effects of climate change, the 95-percentage method could lead to bias due to being influenced by the absolute amount of pollen released during a season. However, in the present study, the 95-percentage method was solely used to define convenient periods within the pollen seasons for the purpose of generating random missing data, rather than for climate-related analyses. This has now been discussed in the manuscript (Page 6, lines from 128 to 129 of the tracked manuscript):

“This method was solely used to define convenient periods within the pollen seasons to generate random missing data.”

2. Line 136: Graminaceae is the former name of the grass family. I recommend using Poaceae everywhere in the manuscript instead.

**Reply**: as suggested by the Reviewer, we have replaced the “Graminaceae” name with the “Poaceae” name in the text and figures.

3. Line 247: correctly: “For both pollen types”, instead of “For both pollens”

**Reply**: as suggested by the Reviewer, we have modified the words.

4. Line 341: correctly: “with”, instead of “whit”

**Reply**: we thank the Reviewer for reporting the typo error. We have corrected it.

Reviewer: 3

Comments to the Author

The manuscript entitled ”A new method based on physical patterns to impute aerobiological datasets” by Tagliaferro et al. presents a novel method to handle missing values in the monitorization of bioaerosols, in comparison to the current method of managing missing data, while also analyzing which factors influence the accuracy of these methods. For this, the authors use pollen data from two different pollen species from two different monitoring stations. The topic of the paper is highly relevant in the context of worldwide increasing allergy prevalence and the influence of climate change on the distribution of respiratory allergens. The paper is overall well written explaining the methods employed for the data comparison.
There are a few minor comments regarding the manuscript.

**Reply**: we thank the Reviewer for their positive view and suggestions for improving our manuscript. We have answered the Reviewer's question, included additional text, and corrected the errors.

We hope that, after the revision, the manuscript will be considered suitable for publication.

1. Could the authors add some information regarding what pollen genera are comprised within the examined Graminaceae pollen and discuss how this could influence the accuracy of the presented methods since they mention a lower variability in this pollen type and a longer pollen season?

**Reply**: we thank the Reviewer for the question. It is challenging to identify specific genera within the Graminaceae family under a microscope, as Italy hosts more than 120 genera and 400 species (D’Amato et al., 2007, Allergy). This broad diversity results in an extended pollen season. Furthermore, the abundance of pollen-producing plants causes pollen to be persistently present in the atmosphere at high concentrations, thus reducing day-to-day variability (i.e., sharp peaks of high pollen concentrations). This information has been added to the Discussion, as suggested by the Reviewer (Page 16, lines from 361 to 363 of the tracked manuscript):

“The abundance of pollen-producing plants within this family, comprising over 120 genera in Italy, leads to high atmospheric pollen levels persisting over extended periods, thereby reducing day-to-day variability and smoothing peaks in pollen concentrations.”

2. Line 120: The authors mention analyzing Alnus and Poaceae pollen, whereas throughout the manuscript they use Graminaceae pollen

**Reply**: as suggested by the Reviewer, we have replaced the “Graminaceae” name with the “Poaceae” name in the text and figures.

3. Line 341 – typo probably ”with” rather than ”whit”

**Reply**: we thank the Reviewer for reporting the typo error. We have corrected it.
